# Spontaneity of nuclear fusion: a qualitative analysis via classical thermodynamics

**DOI:** 10.12688/openreseurope.13738.1

**Published:** 2021-06-11

**Authors:** Silvano Tosti

**Affiliations:** 1 Dept. of Fusion and Technology for Nuclear Safety and Security, ENEA C.R. Frascati, Via E. Fermi 45, Frascati, 00044, Italy

**Keywords:** nuclear reactions thermodynamics, nuclear fusion, nuclear fission, magnetic confinement fusion

## Abstract

**Background:** So far the feasibility of nuclear reactions has been studied only through the evaluation of the reaction rate, which gives us information about the kinetics, while the thermodynamic analysis has been limited to evaluations of the change in enthalpy without any consideration of the change in entropy.

**Methods:** This work examines the thermodynamics of nuclear fusion reactions through a simplified approach. The analysis introduces the thermodynamic study of fission and fusion reactions through their comparison with a chemical process.

**Results:** The main result is that fission reactions are always spontaneous (ΔG < 0) since a lot of energy is released in the form of heat and the system moves spontaneously towards a more disordered state. In contrast, fusion reactions are spontaneous only when the enthalpic contribution of the change in Gibbs free energy overcomes the entropic contribution. This condition is verified when the temperature of the process is below a characteristic value T*, calculated as the ratio between the energy corresponding to the mass defect and the change of entropy of the fusion reaction.

**Conclusions:** Due to the unavailability of data related to entropy changes in fusion reactions, only a qualitative thermodynamic analysis has been carried out. Through such analysis, the influence of the operating conditions over the spontaneity of fusion processes has been discussed. The final considerations emphasize the role of the thermodynamics analysis that should be implemented in the current studies that, so far, have been mainly based on the assessment of the reaction rate and exothermicity of fusion reactions.

## Plain language summary

The manuscript presents a thermodynamic analysis of nuclear processes, both fusion and fission. So far, the feasibility of these processes has been based on the evaluation of the reaction rate (i.e. the probability of reaction) and the (huge) amount of energy released. However, no evaluation of the entropy change that occurs in a nuclear reaction has been made.

This thermodynamic analysis is focused on the different behavior of these nuclear reactions: fusion processes, where light atoms fuse to form a heavier nucleus, should proceed with a negative change of entropy (the system moves towards a more ordered state), while fission processes, in which heavy nuclei split into smaller fragments, evolve with a positive change in entropy. Consequently, fission reactions are always spontaneous while fusion reactions require more accurate evaluation to establish their level of spontaneity. In particular, in this work the operating conditions that promote the spontaneity of the fusion processes are discussed and future approaches are suggested for an accurate evaluation of their entropic change.

## Introduction

The realization of a power plant producing electricity from nuclear fusion is very challenging, although a robust worldwide research and development (R&D) program has been on-going for many years. Since the 1950s, the achievement of the controlled release of fusion energy on a large scale was considered a truly permanent solution to mankind’s expanding need for energy sources ^1^. 

The fusion project is very ambitious: it aims to produce electricity in a substantially CO2-free way, complying with the requirements for environmental sustainability of future energy policies, as well as being inexhaustible and inherently safe ^2– 4^. Despite its outstanding potential impact on the energy sector, nuclear fusion R&D programs have to face very tricky hurdles both in physics and technology ^5– 8^. A significant amount of fusion power has been produced in a controlled way in experimental devices, but the amount is still less than the power injected to heat the plasma ^9^. In an honest comparison of fusion and fission, the development of non-military nuclear fission was realized in a much shorter time and has represented and still represents a significant share among the energy sources. In the case of fission, the scientific phase lasted only about four years, from the final evidence of the uranium fission in December 1938 in Berlin to the realization of the first controlled chain reaction in a reactor in December 1942 in Chicago. The first practical amounts of electricity were generated in 1951 (reactor EBR-I in Idaho) after only five years of total development and construction time, while the first commercial pressurized water reactors, the APS-1 reactor in USSR and the Shippingport reactor in U.S.A., started operation in June 1954 and December 1957, respectively ^10^. The demonstration of uncontrolled thermonuclear fusion goes back to the first H-bomb explosion in November 1952, while in the same period the first projects aimed to develop the scientific and then technological feasibility of fusion power started ^10^. These research projects, through a huge worldwide effort during the last three decades of the 20 ^th^ century with a global budget of more than $1 billion yr ^-1^, led to the realization of several experimental machines ^10^. In November 2006, with the signing of the International Thermonuclear Experimental Reactor (ITER) Agreement by seven members (China, the European Union including Switzerland, India, Japan, Korea, the Russian Federation and the USA), the ITER project was established ^11^. The overall objective of the project is to confirm the feasibility of exploiting magnetic confinement fusion for the production of energy for peaceful purposes by providing an integrated demonstration of the physics and technology required for a fusion power plant ^3, 11^.

This evident difference of progress between fission and fusion R&D programs is supposedly due more to engineering than to physics hurdles ^5^, even though no study has so far investigated the basic thermodynamics of these two nuclear processes, fission and fusion. The feasibility of nuclear reactions has been studied only through the evaluation of the reaction rate, which gives us information about the kinetics, while the thermodynamic analysis has been limited to evaluations of the change in enthalpy without any consideration of the change in entropy.

In this work, the thermodynamics and kinetics of fission and fusion nuclear reactions are approached in analogy to the analysis carried out for chemical processes. Due to the unavailability of chemical-physical properties for many isotopes and nuclear particles, the assessment of the change in entropy for fusion reactions has been conducted via a qualitative analysis, while the change in enthalpy has been calculated as the energy corresponding to the mass defect, a value that is available with enough good approximation for the reactions of interest.

In order to perform a more qualitative thermodynamic analysis, future work could use *ab initio* models for calculating the state functions of isotopes and sub-atomic particles (neutrons, protons, neutrinos, electrons, positrons, etc.) involved in the fusion reactions of interest.

## Study of a chemical process

Classical thermodynamics studies chemical reactions and is used to estimate their spontaneity level. Although in this way no information is given about the kinetics of the reactions, thermodynamics is a powerful tool to evaluate, at equilibrium or near equilibrium conditions, the degree of conversion of a chemical reaction once the operating conditions, namely pressure and temperature, are fixed. A thermodynamic analysis relies on the assessment of state functions, whose definition and detailed description can be found in the huge literature available around basic thermodynamics ^12^. Hereafter, the following state functions will be considered:

- G the Gibbs free energy, J mol ^-1^

- H the enthalpy, J mol ^-1^

- S the entropy, J K ^-1^ mol ^-1^.

The assessment of the change of these state functions (ΔG, ΔH and ΔS) for reversible processes allows prediction of when a reaction proceeds spontaneously (ΔG < 0) and then calculation of, at steady-state or near steady-state, the fraction of reactants converted to products. As an important property of the state functions, it is possible to extend the results of the thermodynamic calculations to any process, even if it is performed in an irreversible way. It is noteworthy to recall as well that, once an initial and final state have been defined, a thermodynamic analysis is uniquely determined and that the change of the state functions (between these initial and final states) does not depend on the intermediate states or the reaction pattern followed.

The relationship:


ΔG=ΔH-TΔS(1)

is useful to evaluate the change in Gibbs free energy from the change in enthalpy and entropy. For a chemical reaction, ΔH and ΔS can be calculated from the values of the state functions of pure elements and the chemical-physical properties of the compounds involved in the reaction. To do this, a lot of databases are available and several calculation tools make this kind of study relatively easy and quick.

An effective example to introduce the comparison with fusion reactions is the reaction between hydrogen and oxygen to form water:


$${\text{H}}_{\text{2}}+\text{0.5}\;{\text{O}}_{\text{2}}={\text{H}}_{\text{2}}\text{O}\;(2)$$

**Figure 1.**
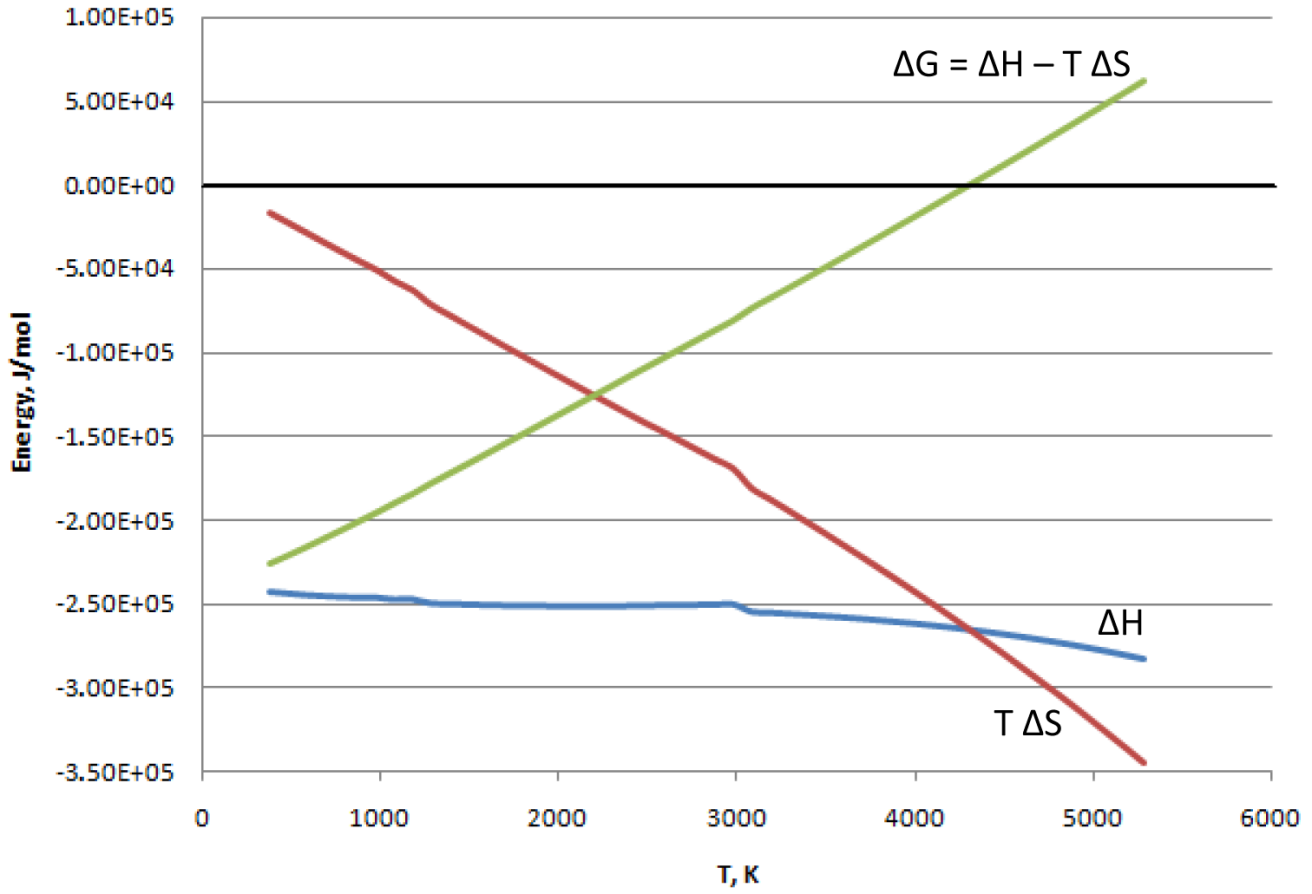
Reaction ( 2): change of state functions vs. temperature.

This reaction takes place with a reduction in the number of moles (1.5 moles react to produce 1 mole of water). Accordingly, it results that ΔS < 0 because the system moves towards a more ordered state while, as is well known, this reaction is very exothermic with negative ΔH values (around 250 kJ mol ^-1^). As represented in Figure 1, calculated at gas phase for the reaction ( 2) by the software Asther ver. 7.12.4 (for a free alternative see FactSage version 8.1), the change in Gibbs free energy starts at low temperatures from negatives values when |ΔH| >> |TΔS|. Then ΔG increases with the temperature as the term |TΔS| grows. The result is that below 4000 K, when ΔG is negative, the reaction proceeds spontaneously. At temperatures higher than 4000 K, it results in ΔG > 0, meaning that the reverse reaction (water splitting to produce hydrogen and oxygen) is promoted.

**Figure 2.**
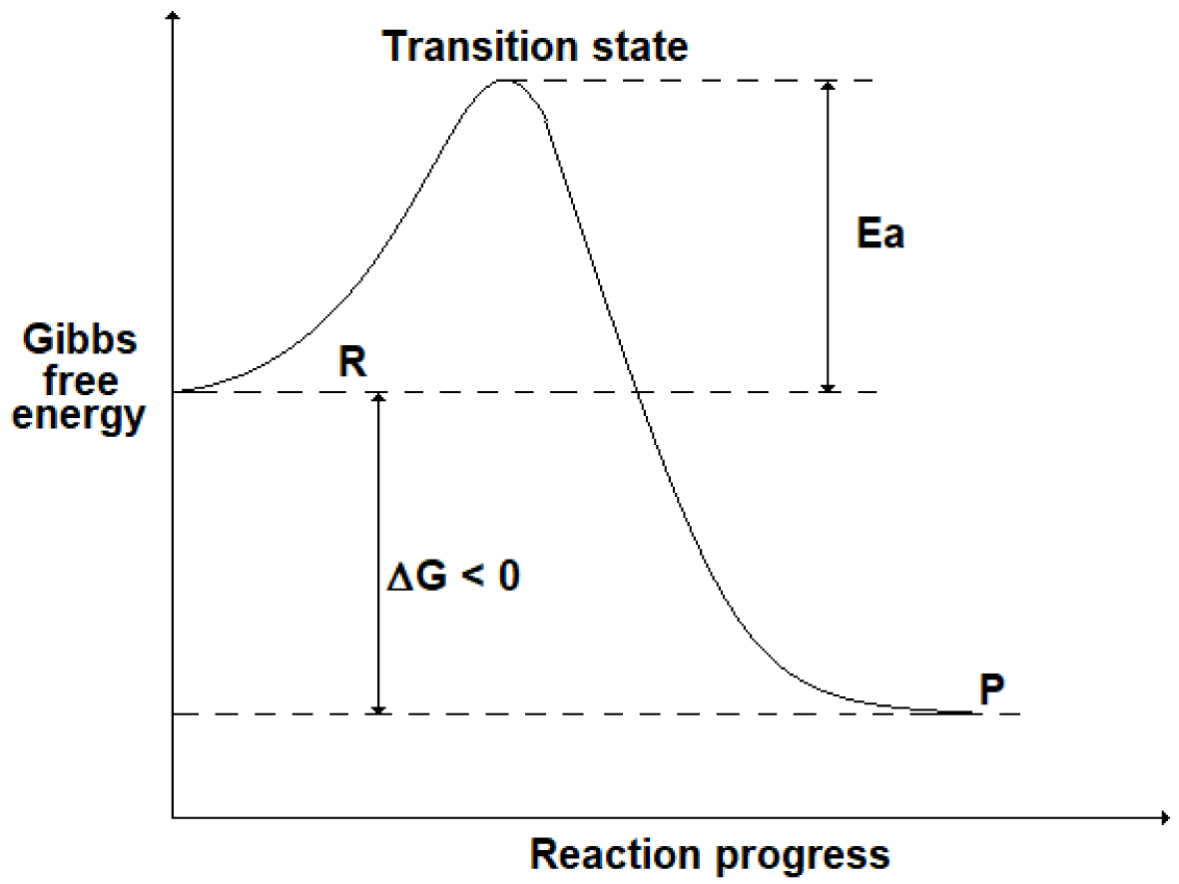
Progress of a chemical reaction and the energy barrier (activation energy, Ea) corresponding to a transition state (TS).

Although the thermodynamic analysis allows us to foresee when a reaction occurs spontaneously, no information is given about its reaction rate, i.e. the speed at which a chemical reaction takes place. For instance, in some cases it can be verified experimentally that a spontaneous reaction (ΔG < 0) proceeds very slowly, making the process studied unfeasible. To explain the kinetics of a chemical reaction, it is useful to introduce the presence of an energy barrier (activation energy, Ea) that corresponds to a “transition state” due to the formation of intermediate compounds: this is shown in Figure 2 where R stands for the initial state (reactants) and P for the final state (products). Even if ΔG < 0 (i.e. G _R_> G _P_), the presence of a high Ea could slow down the reaction progress.

In practice, the higher the energy barrier, the slower the reaction. The use of catalysts is aimed to change the reaction pattern towards the formation of intermediate compounds to which corresponds a lower activation energy and then a higher reaction rate. In particular, the reaction rate (moles of reactants that react per volume and time unit) is defined as the product of the rate constant by the concentrations of the reactants. The rate constant (k, arbitrary units), in turn, can be written:


$$k=A\;e^{-\;\frac{Ea}{R\;T}}\;(3)$$

where A (arbitrary units) is a pre-exponential factor, Ea (kJ mol ^-1^) is the activation energy, R is the molar gas constant (8.31 J K ^-1^ mol ^-1^) and T (K) is the absolute temperature. The higher temperature, the higher the value of k and the faster the reaction. In fact, an increase in temperature corresponds to an increase in the average molecular kinetic energy according to the Maxwell–Boltzmann distribution and this means that more reactant atoms and molecules are able to get over the energy barrier.

## Study of nuclear fusion and fission reactions

Currently, the feasibility of fusion and fission reactions is established by the assessment of their degree of exothermicity (Q-value) and the reaction rate. The thermodynamic analysis of nuclear reactions has so far been limited to the evaluations of the change in enthalpy without any consideration about the change in entropy. However, the kinetic analysis aimed to assess the reaction rate is carried out in a similar way by chemical and nuclear processes as is described in the following.

As previously discussed for chemical processes, basic information about the spontaneity of the process can be definitively provided by thermodynamic analysis considering both the change in entropy and enthalpy. Such an analysis is expected to put in evidence any difference between fusion and fission.

### Change in enthalpy and reaction rate of fusion and fission reactions

Hereafter the evaluation of the Q-value (that corresponds to the change in enthalpy) and of the reaction rate, two parameters presently evaluated to establish the feasibility of a nuclear process, is described.

Nuclear reactions where the total mass of final products is smaller than that of the reactants are exothermic and an amount of energy corresponding to the mass defect is released according to the Einstein’s equation ^13^:


$$\text{E}=\text{Δm}\;{\text{c}}^{\text{2}}\;(4)$$

where Δm is the mass defect and c the speed of light.

When applied to a nucleus, the equation means that the mass of the nucleus is lower of the sum of the masses of its protons and neutrons. Energy corresponding to this mass defect is the binding energy (B) needed to dissociate the nucleus into its components (protons and neutrons).

**Figure 3.**
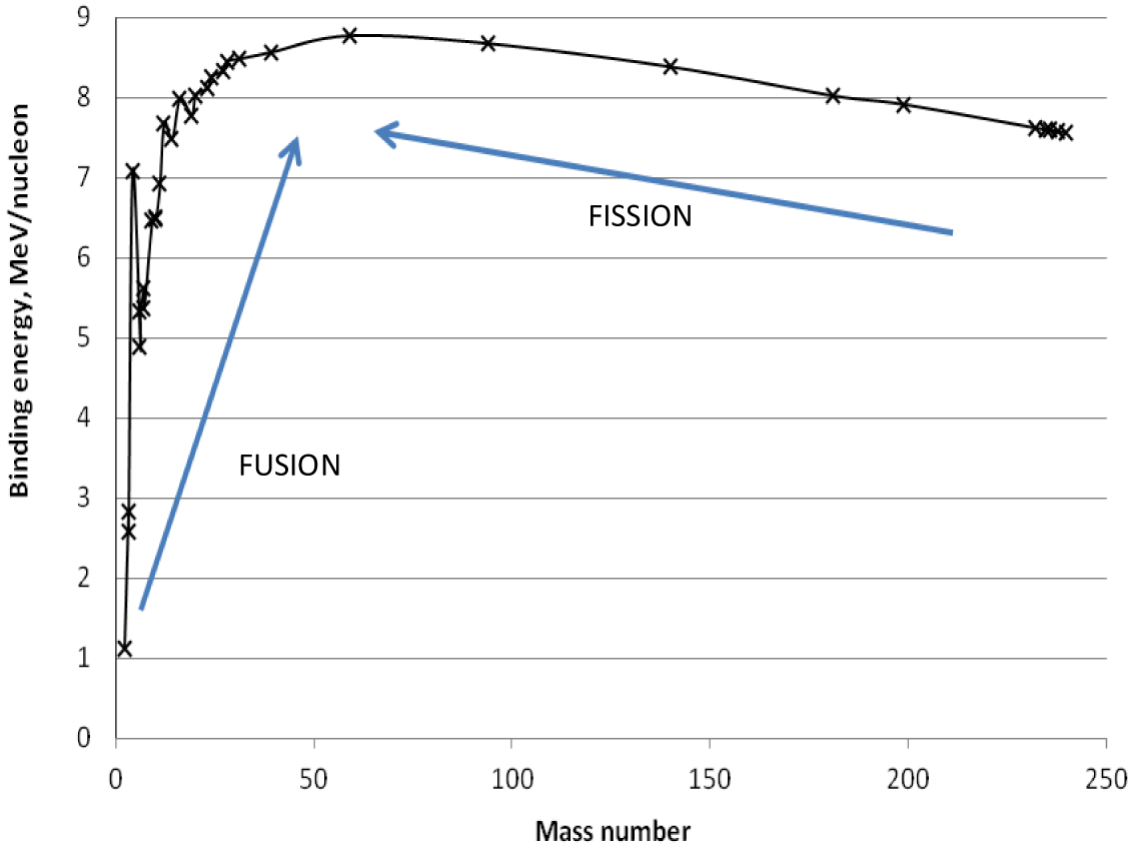
Average binding energy (B) per nucleon (neutron or proton) vs. the mass number (A).

The average binding energy per nucleon (neutron or proton) is characteristic for each element: its behavior vs. the mass number A is reported in Figure 3. Since exothermic nuclear reactions take place when the final products have a B/A larger than the reagents, the maximum B/A at around A = 56 identifies two reactions among the elements: i) light atoms (with A < 56) merge to form a heavier nucleus (i.e. fusion reactions take place), and ii) heavy nuclei (with A > 56) split into smaller fragments (i.e. fission reactions occur).

In magnetic-confinement devices studied for producing energy in a future reactor, deuterium and tritium react to give helium and a neutron:


2+3=4+1(5)

This reaction, occurring at relatively low pressure (few bars) and around 1.5 × 10 ^8^ K, has been selected since it exhibits a very high reaction rate, as will be discussed further on.

In another example, the fusion reactions taking place in the Sun are founded on the proton-proton cycle, for which the overall reaction is:


41=4e+2e++2v(6)

This process based on the weak interaction takes advantage of the very high pressure caused by the gravitational forces present in the Sun (about 1.5 × 10 ^11^ bar) and occurs at temperatures of around 1.5 × 10 ^7^ K.

As an example of fission, the reaction between a nucleus of uranium-235 and a thermal neutron is considered:


235+1=236=141a+92r+31(7)

Both nuclear fission and fusion are very exothermic, the reaction energy, called the Q-value, is the difference between the nuclear rest masses on the initial side and on the final side. It is defined as positive for exothermal reactions and negative for endothermal reactions, opposite to the similar expression in chemical thermodynamics. By assuming that in these nuclear reactions practically all the energy is in the end released as heat, the absolute change in enthalpy, regardless of the different sign conventions, correspond to the Q-value and then to the energy related to the mass defect:


$$|\text{ΔH}|=\text{Q-value}=\text{Δm}\;{\text{c}}^{\text{2}}\;(8)$$

For both fission and fusion, these values of energy are of the order of 1 MeV per nucleon, well above those of the chemical reactions that are ~ 1 eV per nucleon.

The second parameter used to establish the feasibility of a nuclear reaction is the reaction rate, given by the number of reactions per unit time and per unit volume. It is proportional to the cross section that has the dimension of an area and is a measure of the probability that a nuclear reaction will occur. In the case of a nuclear fusion reaction, two positively charged nuclei must come into contact by winning the repulsive Coulomb force. The consequent force field produces a barrier of potential of the order of 1 MeV at the distance corresponding to the nuclear radius while, below such a distance, the nuclear attractive forces prevail ^13^. Although classical mechanics foresees that only nuclei with energy exceeding the potential barrier can react, quantum mechanics allows for tunneling through a potential barrier of finite extension. Gamow’s theory through the cross section links the probability of fusion to the tunneling of the Coloumb barrier:


$$\sigma (\varepsilon )=\frac{S(\varepsilon )}{\varepsilon }\;e^{\left(-\;\sqrt{\frac{\varepsilon_{G}}{\varepsilon }}\right)}\;(9)$$

where σ(ε) is the cross section (arbitrary units) for reacting particles of energy ε (arbitrary units), S(ε) (arbitrary units) is a factor weakly varying as a function of the energy and ε _G _is the Gamow energy that, in practice, acts as an energy barrier. Specifically, ε _G_ is given (here in keV) by the expression:


$$\varepsilon_{G}=986.1\;Z_{1}^{2}Z_{2}^{2}\;A_{r}$$

where Z _1_ and Z _2_ are the atomic mass of the two reacting elements and A _r_ the ratio between the reduced mass (m _1_ × m _2_ /(m _1_ + m _2_)) of the two reacting elements and the proton mass. This means that the chance of tunneling decreases rapidly with the atomic number and mass; for this reason, fusion reactions of interest for energy production on Earth involve the lightest nuclei, namely hydrogen isotopes and helium ^13^.

Among the potential fusion reactions of light atoms, the reaction of deuterium and tritium ( 5) has been selected since it exhibits the highest cross section (i.e. the highest reaction probability) at the lowest energy: 5.0 barn at 64 keV (about 7.42 × 10 ^7^ K), as can be seen in Table 1.

**Table 1.** Cross sections at center of mass energy of 10 and 100 keV, maximum cross sections (σ _MAX_) and location of the maximum (ε _MAX_) of fusion reactions ^13^.

Reaction	σ at 10 keV (barn)	σ at 100 keV (barn)	σ _max_ (barn)	ε _max_ (keV)
2+3=4+1	2.72 × 10 ^-2^	3.43	5.0	64
2+2=3+1	2.82 × 10 ^-4^	3.3 × 10 ^-2^	0.096	1250
2+2=3+1	2.78 × 10 ^-4^	3.7 × 10 ^-2^	0.11	1750
3+3=4+21	7.90 × 10 ^-4^	3.4 × 10 ^-2^	0.16	1000
2+3=4+1	2.20 × 10 ^-7^	0.1	0.9	250
1+1=2+e++γ	3.6 × 10 ^-26^ *	4.4 × 10 ^-25^ *	-	-

* Values estimated.

The second most probable reaction is:


2+3=4+1(10)

which has a lower cross section (0.9 barn) and requires the achievement of much higher energies/temperatures (250 keV, about 2.9 × 10 ^9^ K).

In comparison, the proton-proton reaction (part of the proton-proton cycle) is extremely slow and has cross sections of the order of 10 ^-25^ barn.

By recalling that in plasma physics the energy of the particles is linked to their temperature through the Boltzman constant (1 keV ≈ 1.13 × 10 ^9^ K), it is noteworthy to consider the analogy between the kinetic expressions ( 3) and ( 9) which, for chemical and nuclear processes respectively, relate the reaction rate to the capability of the reacting species to overcome an energy barrier (E _a_ and ε _G_) on the basis of their temperature (i.e. energy level).

### Simplified thermodynamic analysis of fusion and fission reactions

In contrast to the approach adopted so far, which establishes the feasibility of nuclear processes only based on kinetic evaluations (assessment of reaction rate) and on the change in enthalpy, in the following discussion the change in Gibbs free energy and then the level of spontaneity of these reactions is calculated by also considering the entropic contributions.

Since no database is available for many isotopes and nuclear particles, the assessment of ΔH and ΔS can be done only via a qualitative analysis.

As described in the previous section, the change in enthalpy can be evaluated with good approximation by the Q-value and then by the energy corresponding to the mass defect.

For the change in entropy ΔS, it is convenient to represent the nuclear reaction as similarly as possible to a chemical reaction by correctly choosing the initial and the final state of the system and neglecting the presence of sub-atomic and other light particles (neutrons, protons, etc.). In this respect, the reaction ( 5) expressed in a classical thermodynamic form becomes:


D+T=He+Q(11)

where the initial state consists of two atoms, deuterium (D) and tritium (T), while the final state is given by an atom of helium plus the heat Q released by the process and corresponding to the Q-value or the absolute change in enthalpy. In particular, this final state corresponds to the production of helium and the release of heat (Q), according to what happens in the magnetic fusion devices at the level of blanket systems where the energy carried by the neutrons is changed into heat.

According to this perspective, the change in entropy of a fusion reaction (ΔS _FUS_) is estimated to be negative since more light atoms merge to form a heavier nucleus and thus we can expect an increase in the order of the system. In fission, heavy nuclei split into smaller fragments and therefore, we assume that ΔS _FIS_ is positive.

**Figure 4.**
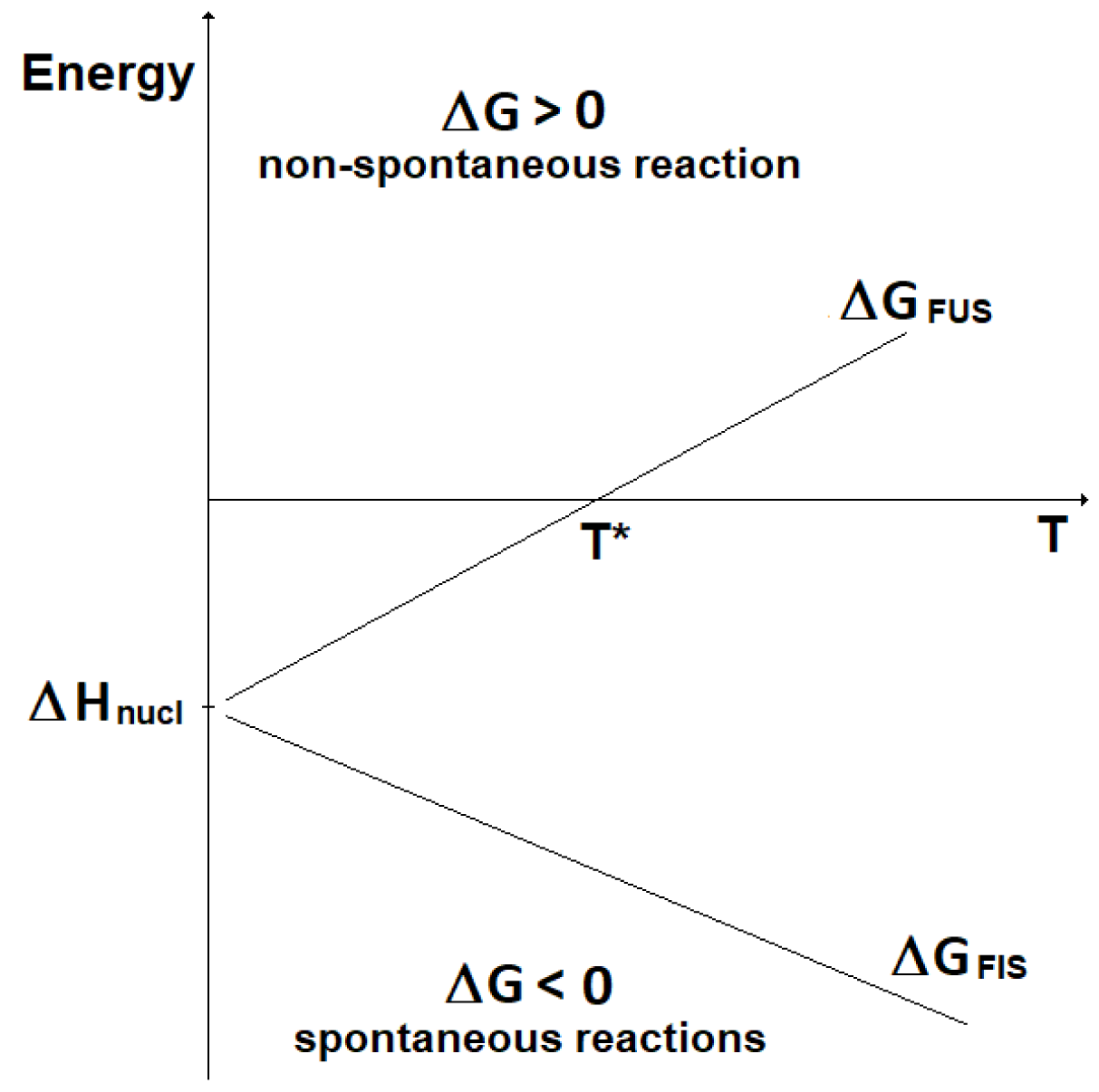
Change in Gibbs free energy for fission (ΔG _FIS_) and fusion (ΔG _FUS_) reactions vs. temperature (qualitative).

Again, using a qualitative approach, the change in enthalpy ΔH assumes approximately the same value for fission and fusion (ΔH _FIS_ ≈ ΔH _FUS_ ≈ ΔH _nucl_), while the change in entropy (ΔS) can be considered almost constant with the temperature. Accordingly, the change in Gibbs free energy for the two kinds of nuclear reactions is represented as reported in Figure 4. Here, it can be seen that fission reactions are spontaneous anyway (ΔG < 0), since both the enthalpic and the entropic terms give a negative contribution to the Gibbs free energy. In fact, in fission a lot of energy is released in the form of heat and, in addition, the system moves spontaneously towards a more disordered state. In contrast, fusion reactions are spontaneous (ΔG < 0) only when the enthalpic contribution overcomes the entropic contribution. In practice, to guarantee the spontaneity of a fusion reaction the absolute value of its entropy change has to be, through its product with the temperature, smaller than the energy corresponding to the mass defect. The term |ΔS _FUS_| _spont_ is introduced:


$${\left|\text{Δ}S_{FUS}\right|}_{spont}\approx \frac{\text{Δ}m\;c^{2}}{T}\;(12)$$

In order to have spontaneous reactions (i.e. ΔG < 0), the change in entropy has to be |ΔS| < | *ΔS*_*FUS*_| _*spont*_.

The lower the temperature, the most spontaneous the fusion reaction. It results that fusion reactions are spontaneous for temperatures below the value T*:


$$T^{*}=\frac{\Delta H}{\Delta S}\;(13)$$

Under the assumptions done, this can be written:


$$T^{*}\approx \frac{\text{Δ}m\;c^{2}}{|\text{Δ}S_{FUS}{|}_{spont}}\;(14)$$

A first consideration coming from this basic analysis is that the reaction ( 5), taking place at temperatures around 1.5×10 ^8^ K, will proceed spontaneously when the absolute entropy change is lower than the limit value | *ΔS*_*FUS*_| _*spont*_ = 1.17×10 ^-1^ eV K ^-1^. For the reaction ( 6) proceeding at 1.5 × 10 ^7^ K, the limit value of the entropy change, | *ΔS*_*FUS*_| _*spont*_, rises up to 9.33 × 10 ^-1^ eV K ^-1^, is almost an order of magnitude higher than that of reaction ( 5). In this sense, the proton-proton cycle exhibits a level of spontaneity far above that of the deuterium-tritium reaction.

**Figure 5.**
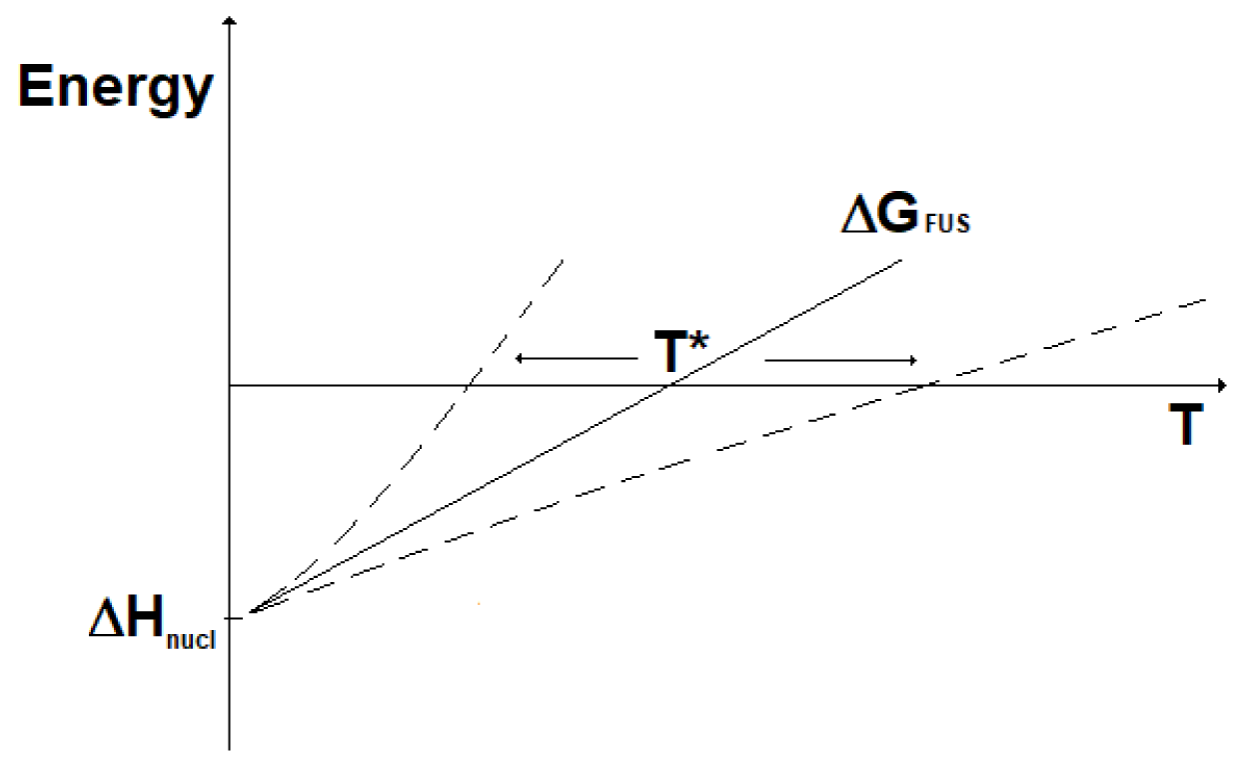
Fusion reactions: impact on T* of ΔG deviation from linearity.

Further considerations are extracted along with the simplified assumption that the change in entropy of a fusion reaction may be of the same order of magnitude of a chemical reaction. For reaction ( 2) and other similar chemical syntheses (e.g. the hydrogen iodide formation: H _2_ + 2I = 2HI), the absolute value of the change in entropy, |ΔS|, is estimated of the order of 10 ^2^ J mol ^-1^ K ^-1^, corresponding to a value of about 1.0 ×10 ^-3 ^eV K ^-1^ per particle. Clearly, such a calculation is based on a number of approximations and, therefore, does not allow extraction of any practical conclusion about the spontaneity of the fusion reactions above-considered. However, these results can suggest how to approach the future study of nuclear fusion reactions. With reference to the deuterium-tritium reaction, the very high reaction temperature and the particular matter state (plasma) could involve deviation from the linearity of free Gibbs energy and lead to values of T* different (either smaller or larger) to those assessed by this simplified approach, see Figure 5. Other deviation from the linearity of the change in free Gibbs energy could be due to the dependence on temperature of the change in entropy, which in this qualitative analysis has instead been considered constant.

In particular, the way to heat the plasma in the fusion devices running the deuterium-tritium reaction could impact on its entropy content and, therefore, it could be worth identifying the heating power systems capable of positively influencing the spontaneity of the fusion reaction.

From this preliminary analysis a couple of conclusions can be extracted:

- In contrast to fission, which is always spontaneous, for fusion the reaction probability assessment should be integrated with a thermodynamic analysis in order to establish its spontaneity level in relation to the operating conditions proposed.

- The temperature affects a fusion reaction differently through thermodynamics (increasing the temperature of the reaction reduces its spontaneity) and kinetics (increasing the T, at least until a given value, increases the reaction rate).

Furthermore, it should be evaluated whether these conclusions about the thermodynamics and kinetics of fusion reactions could allow the controversial “cold fusion” experiments to be revisited ^14^. Following the indications of this analysis, thermodynamics should positively affect fusion carried out at low temperatures (≈ 10 ^2^ K). On the other hand, the low temperature would involve very slow kinetics that could be responsible for the poor reproducibility of these experiments and make any application unfeasible.

### Future work

As discussed, the change in enthalpy of a nuclear reaction can be calculated as the energy corresponding to the mass defect. Since this value is available with enough good approximation for the reactions of interest, it remains to see how it is possible to do a more quantitative assessment of the change of entropy.

For chemical reactions, databases exist with values of the state functions and physical-chemical properties of elements and compounds, which allow calculation of the reaction ΔS. However, these data are not available for the isotopes involved in nuclear reactions. In addition, it is necessary to understand the role in the thermodynamic analyses of the sub-atomic particles involved (neutrons, protons, neutrinos, electrons, positrons, etc.) and establish their contribution to the state functions calculation. As already done for molecules, these assessments could be carried out via *ab initio* quantum chemistry methods that exhibit good accuracies of around 1 kcal mol ^-1^ ^15– 17^. For nuclear systems and sub-atomic particles in particular, most *ab initio* methods should be re-designed for calculations at non-zero temperatures in order to access nuclear thermodynamics. In many-body perturbation theory (MBPT), diagrammatic expansions are used to calculate bulk thermodynamic properties ^18, 19^ or self-consistent Green’s functions, which provide non-perturbative solutions of the finite temperature system ^20, 21^. Beside these methods, lattice effective field theory has been recently used in conjunction with Monte Carlo simulations for *ab initio* calculations of the thermodynamics of nuclear systems ^22^.

## Conclusions

In analogy with the study of chemical processes based on the assessment of the state functions, the thermodynamics of a nuclear reaction can establish its level of spontaneity.

A preliminary thermodynamic analysis of nuclear reactions has shown the different behavior of fission and fusion, which proceed with a negative and positive change in entropy, respectively. Fission reactions are always spontaneous (ΔG < 0), while the fusion ones are spontaneous only for temperatures below a critical value (T*). Such a condition is verified when the enthalpic contribution overcomes the entropic contribution, i.e. when the product of its entropy change with the temperature (T |ΔS _FUS_|) is smaller than the energy released by the reaction (the Q-value of the nuclear reaction).

These preliminary considerations emphasize the role of the thermodynamics analysis for the study of the fusion reactions that are of interest for application in magnetic confinement devices that, so far, have been selected on the basis of feasibility criteria evaluating only their reaction probability (i.e. reaction rate) and exothermicity.

In conclusion, this work would suggest the need for a more detailed thermodynamic analysis of fusion reactions, which could make some aspects related to the influence of operating conditions on spontaneity clearer. Future studies could take advantage of the recent developments in *ab initio* models for calculating the state functions of isotopes and sub-atomic particles.

## Data availability

All data underlying the results are available as part of the article and no additional source data are required.
