# Supplementary material for: Spontaneity of nuclear fusion: a qualitative analysis via classical thermodynamics
Source: Open Res Eur. 2021 Oct 12;1:67. Originally published 2021 Jun 11. [Version 3] doi: 10.12688/openreseurope.13738.3 (PMC10446017; doi:10.12688/openreseurope.13738.3)
Supplement: Peer review history

# Peer review: Reviewer response for version 3

**DOI:** 10.21956/openreseurope.15307.r27789

**Published:** 2021-10-13

**Authors:** Viorel Badescu

**Affiliations:** 1 Candida Oancea Institute, Polytechnic University of Bucharest, Bucharest, Romania

Approved

Is the study design appropriate and does the work have academic merit?

Yes

Is the work clearly and accurately presented and does it cite the current literature?

Yes

If applicable, is the statistical analysis and its interpretation appropriate?

Partly

Are all the source data underlying the results available to ensure full reproducibility?

Partly

Are the conclusions drawn adequately supported by the results?

Partly

Are sufficient details of methods and analysis provided to allow replication by others?

Partly

Reviewer Expertise:

NA

I confirm that I have read this submission and believe that I have an appropriate level of expertise to confirm that it is of an acceptable scientific standard.

# Peer review: Reviewer response for version 2

**DOI:** 10.21956/openreseurope.15122.r27546

**Published:** 2021-09-21

**Authors:** Baudilio Coto García

**Affiliations:** 1 Department of Chemical, Energy and Mechanical Technology, King Juan Carlos University, Madrid, Spain

The paper reports a thermodynamic analysis for nuclear reactions, including eventual entropic effects, usually not considered in this kind of process. Several assumptions are made and only qualitative analysis is intended. Simultaneous descriptions of energy, entropy, and kinetic effects are considered. Future studies are proposed to complete the presented work.

 Some changes are suggested according to next points:

- ΔG<0 is the spontaneity criteria when T and P are constant. It should be clearly stated that the conditions of the fusion reactions discussed throughout the work meet these conditions.
- Along with the paper, several comparisons are made to conventional chemical reactions, and even it is stated for entropy that |ΔS _fus_|~|ΔS _r_|. However, |ΔH _fus_|>>|ΔH _r_|, and consequently, it could be that |ΔH _fus_|>>T|ΔS _fus_| and entropy effect could be negligible, ie. that slope in Fig 4 was very low. Even when work is only a qualitative analysis, the expected magnitude orders should be detailed to clarify such points.
- G has to be named “Gibbs energy” in the place of “Gibbs free energy”.
- In the activation energy, E _a_, the “a” has to be a subscript.
- Equation (3) has to be named the Arrhenius equation.
- k and A in the Arrhenius equation do not have “arbitrary units” but units dependent on the reaction order.

Is the study design appropriate and does the work have academic merit?

Partly

Is the work clearly and accurately presented and does it cite the current literature?

Yes

If applicable, is the statistical analysis and its interpretation appropriate?

Not applicable

Are all the source data underlying the results available to ensure full reproducibility?

Partly

Are the conclusions drawn adequately supported by the results?

Partly

Are sufficient details of methods and analysis provided to allow replication by others?

Yes

Reviewer Expertise:

Thermodynamic

I confirm that I have read this submission and believe that I have an appropriate level of expertise to confirm that it is of an acceptable scientific standard.

# Peer review: Reviewer response for version 2

**DOI:** 10.21956/openreseurope.15122.r27516

**Published:** 2021-09-21

**Authors:** Gianfranco Caruso

**Affiliations:** 1 DIAEE Department, Sapienza University of Rome, Rome, Italy

A preliminary thermodynamic analysis of nuclear reactions is presented in the paper, providing some useful introductory aspects for the reader, mainly in establishing the spontaneity of fission and fusion nuclear reactions.

 About future work, the author could also refer to an interesting paper published in 2018, (Palazzo, P.  "Fission and fusion nuclear reactions second law analyses based on exergy method" ) where the thermodynamic entropy calculation has been carried out in the perspective of the Second Law analysis based on the exergy method, adopted to calculate entropy property variations in fission and fusion nuclear processes. In that work, irreversibilities in the physics of elementary particles have also been discussed. It is an interesting approach, that could provide a valid basis for assessing the entropy variation and could be cited in the "future work" section of the present paper.

Is the study design appropriate and does the work have academic merit?

Yes

Is the work clearly and accurately presented and does it cite the current literature?

Yes

If applicable, is the statistical analysis and its interpretation appropriate?

Not applicable

Are all the source data underlying the results available to ensure full reproducibility?

Yes

Are the conclusions drawn adequately supported by the results?

Yes

Are sufficient details of methods and analysis provided to allow replication by others?

Yes

Reviewer Expertise:

Nuclear Engineering

I confirm that I have read this submission and believe that I have an appropriate level of expertise to confirm that it is of an acceptable scientific standard.

## References

[1] : Fission and Fusion Nuclear Reactions Second Law Analyses Based on Exergy Method. *International Journal of Thermodynamics* .2018;21(4) : 10.5541/ijot.452516 221-229 10.5541/ijot.452516

# Peer review: Reviewer response for version 2

**DOI:** 10.21956/openreseurope.15122.r27335

**Published:** 2021-08-06

**Authors:** Viorel Badescu

**Affiliations:** 1 Candida Oancea Institute, Polytechnic University of Bucharest, Bucharest, Romania

I have read the up-dated version on the ms by Dr Tosti. It looks fine for me. I do not have further comments.

Is the study design appropriate and does the work have academic merit?

Yes

Is the work clearly and accurately presented and does it cite the current literature?

Yes

If applicable, is the statistical analysis and its interpretation appropriate?

Partly

Are all the source data underlying the results available to ensure full reproducibility?

Partly

Are the conclusions drawn adequately supported by the results?

Partly

Are sufficient details of methods and analysis provided to allow replication by others?

Partly

Reviewer Expertise:

NA

I confirm that I have read this submission and believe that I have an appropriate level of expertise to confirm that it is of an acceptable scientific standard.

# Peer review: Reviewer response for version 1

**DOI:** 10.21956/openreseurope.14814.r27103

**Published:** 2021-06-30

**Authors:** Viorel Badescu

**Affiliations:** 1 Candida Oancea Institute, Polytechnic University of Bucharest, Bucharest, Romania

The theory is based on Eq. (1) which is used as a tool when treating fusion reactions by similarity with usual chemical reactions. The theory is based on many qualitative assumptions since data is not available for the enthalpy and entropy variation for several isotopes and nuclear particles. Replacing these assumptions would require using other questionable assumptions. The section Future Work leaves ways for further improvement. The following two comments should be answered:

1. A problem is defining T in Eq. (1), in case fusion reactions are considered. Is it the usual temperature, related with kinetic energy of molecules and atoms (which can be measured by using a common thermometer, for instance)? Is always the temperature of the nucleons the same with the temperature of the molecule (for instance, at low temperatures such as 10^2 K, considered in the paper? These aspects should be clarified.
2. Notice that thermodynamic state functions such as enthalpy, entropy and Helmholtz free energy have been previously evaluated for nuclear reactions based on the Weisschoff theory of compound nucleus (described in detail in V. Badescu, Int. J. Nuclear Governance, Economy and Ecology, Vol. 2, No. 1, 2008 , 90-112 and in a short version in V. Badescu, Central European Journal of Physics volume 7, 141–146 (2009)). The main difference is that the state functions have been evaluated by using *ab-initio* statistical thermodynamics calculation while in the present paper changes in entropy, enthalpy and Gibbs free energy are estimated qualitatively by using standard thermodynamics and mass defect. Are the two approaches complementary? Is it possible to use them for crosschecking? Comments may be provided on this line.

Is the study design appropriate and does the work have academic merit?

Yes

Is the work clearly and accurately presented and does it cite the current literature?

Yes

If applicable, is the statistical analysis and its interpretation appropriate?

Partly

Are all the source data underlying the results available to ensure full reproducibility?

Partly

Are the conclusions drawn adequately supported by the results?

Partly

Are sufficient details of methods and analysis provided to allow replication by others?

Partly

Reviewer Expertise:

statistical thermodynamics

I confirm that I have read this submission and believe that I have an appropriate level of expertise to confirm that it is of an acceptable scientific standard, however I have significant reservations, as outlined above.

## References

[1] : Radiation exergy: the case of thermal and nuclear energy. *International Journal of Nuclear Governance, Economy and Ecology* .2008;2(1) : 10.1504/IJNGEE.2008.017357 10.1504/IJNGEE.2008.017357

[2] : Exergy of nuclear radiation — a quantum statistical thermodynamics approach. *Open Physics* .2009;7(1) : 10.2478/s11534-008-0136-8 10.2478/s11534-008-0136-8

# Response

**Published:** 2021-07-30

**Authors:** Silvano Tosti

**Affiliations:** 21412, Italy

*Reply to the Reviewer Report from Dr. Viorel Badescu* **(**reviewer comments/questions *,* **author reply** *,* *sentences added in the text* **)**

 The theory is based on Eq. (1) which is used as a tool when treating fusion reactions by similarity with usual chemical reactions. The theory is based on many qualitative assumptions since data is not available for the enthalpy and entropy variation for several isotopes and nuclear particles. Replacing these assumptions would require using other questionable assumptions. The section Future Work leaves ways for further improvement. The following two comments should be answered:

1. A problem is defining T in Eq. (1), in case fusion reactions are considered. Is it the usual temperature, related with kinetic energy of molecules and atoms (which can be measured by using a common thermometer, for instance)? Is always the temperature of the nucleons the same with the temperature of the molecule (for instance, at low temperatures such as 10^2 K, considered in the paper? These aspects should be clarified.

***According to classical therm*odynam *ics, the temperature defined in Eq. 1 is related to the kinetic energy of molecules and atoms (i.e. the usual one). In order to reduce the uncertainties related to the presence of subnuclear particles, when possible the thermodynamic analysis is carried out with reference to states of matter in which nucleons are not present. For instance, the DT reaction is studied through the Eq. 11 that contains only atoms: in its initial state there are one deuterium atom and one tritium atom while the final state is represented by an He ^4^ atom plus an amount of heat corresponding to the energy released by the neutron interaction with the Li-blanket. The comment raised by the reviewer could be relevant when discussing about the “cold fusion” where the definition of the initial and final states and presence of nucleons should better be clarified case by case. In this vein, the author proposes to revise the part of the manuscript discussing about the “cold fusion” as follows:***

*Furthermore, it should be evaluated whether these conclusions about the thermodynamics and kinetics of fusion reactions could allow the controversial “cold fusion” experiments to be revisited [14].* *At low temperature (≈ 10 ^2^ K) the kinetic energy of nucleons could be different from that of atoms and molecules and, therefore, a classical thermodynamic analysis may be applied when the initial and final states of the reactions considered are fixed without the presence of subnuclear particles. In such a case, this analysis shows that thermodynamics should positively affect fusion carried out at low temperatures. On the other hand, the low temperature would involve very slow kinetics that could be responsible for the poor reproducibility of these experiments and make any application unfeasible.*

1. Notice that thermodynamic state functions such as enthalpy, entropy and Helmholtz free energy have been previously evaluated for nuclear reactions based on the Weisschoff theory of compound nucleus (described in detail in V. Badescu, Int. J. Nuclear Governance, Economy and Ecology, Vol. 2, No. 1, 2008 , 90-112 and in a short version in V. Badescu, Central European Journal of Physics volume 7, 141–146 (2009)). The main difference is that the state functions have been evaluated by using *ab-initio* statistical thermodynamics calculation while in the present paper changes in entropy, enthalpy and Gibbs free energy are estimated qualitatively by using standard thermodynamics and mass defect. Are the two approaches complementary? Is it possible to use them for crosschecking? Comments may be provided on this line.

***Thanks to the reviewer for this suggestion. In the revised text, the calculation of thermodynamic functions via statistical approach has been discussed in the section related to the future work. It is planned that next studies will use classical statistical thermodynamics (e.g. Eq. of Tetrode-Sackur) and their results will be compared with entropy calculations based on the literature suggested by the reviewer. The following text has been added at the end of the “Future work” section:***

*Future work is also planned to rely on statistical thermodynamics that allows to calculate the entropy of particles and intermediate compounds involved in nuclear reactions. Statistical analogous of entropy and other thermodynamic functions of atoms and molecules could be evaluated through these methods, e.g. the Sackur-Tetrode equation [12]. In parallel, quantum statistical thermodynamic approach introduced to assess the exergy of nuclear radiations can provide the expressions to calculate the entropy of “compound nucleuses”, intermediate compounds consisting of target nucleuses and the projectiles of nuclear reactions in excited states [23, 24]. Calculations based on these statistical thermodynamic methods and their comparison are expected to provide a more accurate thermodynamic evaluation of fusion reactions.*
